# Effect of sternal closure with biological bone adhesive on pain visual analogue score and serum cytokine

**DOI:** 10.1186/s13019-015-0230-0

**Published:** 2015-03-17

**Authors:** Shahrul Hashim, Leow Yeen Chin, Sivakumar Krishnasamy, Pavai Sthaneswar, Raja Amin Raja Mokhtar

**Affiliations:** 1 Division of Cardiothoracic Surgery, Department of Surgery, University Malaya Medical Centre, 50603 Kuala Lumpur, Malaysia; 2 Department of Pathology, University Malaya, 50603 Kuala Lumpur, Malaysia

**Keywords:** Sternal closure, Biological bone adhesive, Cytokine, Pain

## Abstract

**Objectives:**

Recently a biocompatible bone adhesive was introduced in addition to the sternal wires to expedite sternal union and improve patient recovery. In this study we aim to objectively assess the biomarker of pain in patient who received the biocompatible bone adhesive.

**Methods:**

A total of 62 patients who underwent sternotomy were prospectively randomised to receive either conventional wire closure (CWC); 32 patients or adhesive enhanced closure in addition to sternal wire (AEC); 30 patients. Patients were monitored postoperatively at certain time intervals for incisional pain, serum Interleukin-6 (IL-6) level, analgesia used and postoperative complications. All patients were followed up for 4 weeks.

**Results:**

The post-operative pain scores with coughing were significantly higher in the CWC group at 24 hours and 48 hours. The postoperative IL 6 levels were significantly higher in the CWC group in comparison with the AEC group at 6 hours, 24 hours, and 48 hours. There were no significant differences in term of additional analgesia used. No adverse events from adhesive bone cement were observed during follow up.

**Conclusions:**

Adhesive-enhanced sternal closure resulted in modest reduction of pain confirmed by reduction of pain biomarker. Justification of its routine use requires larger multicentre study.

## Background

Since its introduction in 1957 median sternotomy has been be the most popular incision to access the cardiac structures for open heart surgery [1]. Despite the recent advances in minimal access and robotic cardiac surgery, median sternotomy remains the incision of choice and will probably continue to be popular in the future [2,3]. The complications from median sternotomy include acute and chronic pain, instability, non-union, malunion and infections [4,5]. Each of these complications can vary in severity and can be potentially fatal especially in the case of deep sternal wound infection [6]. Closure techniques with efficient early mechanical stability are very important for the functional recovery and reducing the risk of these complications [7]. Recently a novel biological bone adhesive (Kryptonite, doctors Research Group Inc, Southbury, CT) was introduced to facilitate sternal closure [8,9]. A few studies had demonstrated its safety and effectiveness in term of pain, respiratory function and quality of life [8,10,11]. However majority of these findings especially pain can be subjective especially when measured using currently available visual pain score tools. We therefore aim to compare the utilisation of the biological bone adhesive as an adjunct to the conventional sternal closure versus conventional closure alone in term of an objective pain assessment utilising a serum cytokine as a pain biochemical marker in addition to the standard visual pain score analysis.

## Methods

### Study design and settings

This is a single blinded prospective randomised study conducted in a single centre between July 2012 and December 2012. University Malaya Medical Centre is a large tertiary teaching hospital in Kuala Lumpur Malaysia serving a population of up to 3 million people. The cardiothoracic unit performs up to three hundred fifty adult open heart surgeries per year. The study was funded by the local university research grant. Ethical approval was obtained from the University Malaya Medical Centre Kuala Lumpur Malaysia ethics committee. The patients were randomly assigned to either closure with conventional wire closure (CWC) or adhesive enhanced closure group (AEC). All patients, caregivers and research assistant were blinded to the intervention. The patients received standard postoperative care and standard analgesia protocol. All data were collected by a research assistant and were stored in a secured site at the hospital.

### Study population

Consecutive adult patients over the age of 18 who were undergoing first time elective or semi elective open heart surgery through a median sternotomy were invited for the study. Exclusion criteria were:

- Emergency surgery (defined as surgery within 24 hours of diagnosis)
- At high risk of postoperative bleeding (taking antiplatelet within 5 days of surgery, redo surgery)
- Previous radiotherapy to chest wall
- Preoperative condition which can potentially cause prolonged recovery such as significant respiratory disease, renal failure on dialysis, current used of steroid or current substances abused

### Conduct of study

All patients were randomly assigned to receive either closure with conventional wire closure (CWC group) or adhesive enhanced closure (AEC group). A computer-generated random numbers were used for randomization of subjects into each group. Whereas the surgeons were aware of the allocated intervention or control group, patients, outcome assessors and data analysts were kept blinded to the allocation. The anaesthesia and surgery were performed according to routine standard of care. For sternal closure, all sternums were reapproximatted with 5 figure-of-8 sternal wire size 5. The AEC group received an additional bone adhesive on top of the standard closure. The use of bone wax was prohibited. In the AEC group, the trabeculae of each hemi sternum were cleaned with the provided brush and irrigated with saline solution. The ‘Kryptonite’ bone adhesive was mixed for 5 minutes and following which 5 ml of adhesive was applied as a thin layer to each hemi sternum at the trabeculae interface. Before the adhesive became complete set which was about 8 minutes the two hemi sternum were approximated. An additional 15 minutes of operative time were utilised for the application of the biological bone adhesive. The soft tissue and skin were closed in a routine manner in both groups. Post operatively the cares were identical in both groups. All patients received a standardized postoperative oral analgesia protocol which comprised of oral Paracetamol (PCM) 1 g QID and capsule Tramadol 50 mg TDS. Standard preoperative demographic data of the patients were collected. The primary outcome for this study was post-operative pain measurement. Biochemical marker of pain was measured by monitoring the serum Interleukin-6 (IL 6). The serum IL 6 (pg/ml) was measured preoperatively and at 6 hours, 24 hours, 48 hours and 72 hours postoperatively. IL 6 was measured with a commercially available enzyme linked immunosorbent assay (ELISA). The Visual Analogue Score (VAS) based on Wong-Baker faces pain scale which utilizes of face depictions that range from happy to tearful (0 = none, 10 = worst pain ever) were collected by a research assistant at 24 hours, 48 hours, 72 hours, and day 7 at rest and at coughing postoperatively. Also recorded as secondary outcome were complications following surgery which include death, chest reopen, perioperative MI, renal failure, pneumonia and sternal wound infection. The patients were followed up until 4 weeks following surgery.

### Statistical analysis

Based from the previous study the ability to detect the differences in pain score measurement of 3 (range 0–5.5) between the two groups were used to calculate our sample. We estimated that the enrolment of 50 patients would provide a power of 80% to detect the anticipated difference in primary outcome at a two-sided alpha level of 0.05. To allow for drop out, a total of 62 patients were recruited during the study period.

All analysis was based on intention-to-treat principle. All data were entered and statistical analysis were performed using SPSS for Windows version 15 (SPSS Inc, Chicago, IL). Categorical data were expressed as percentage and analysed using Chi-Square test. Parametric data which were normally distributed were reported as mean +/− standard deviation and analysed with One-Way-Anova using Bonferonni correction to test for significance. Non parametric data which had skewed distribution were analysed using Kruskal-Wallis Test and reported as median. Values of p less than 0.05 were considered significant.

## Results

**Figure 1:**
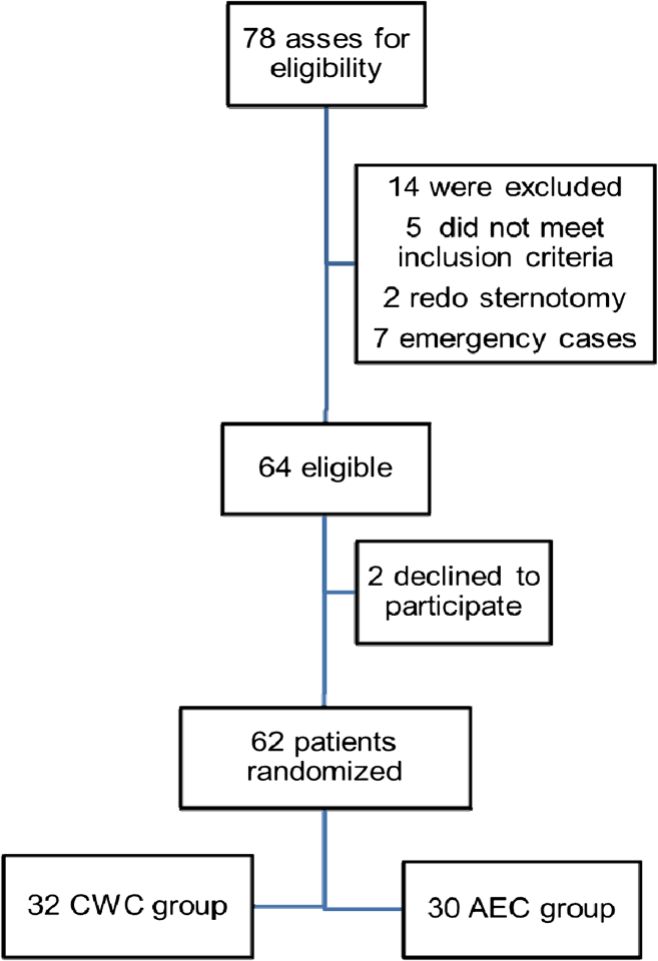
**Patients recruitment flow diagram according to CONSORT guidelines.**

Between October 2012 and December 2012, a total of 78 patients were asses for eligibility into the study (Figure 1). Of the 64 eligible patients 2 refused to participate. The study population demographic demonstrated more male patients with majority receiving a coronary bypass graft surgery (Table 1). The baseline characteristics of the 62 patients in both groups were similar (Table 1). All the coronary artery bypass cases are done using cold blood cardioplegic arrest and cardiopulmonary bypass techniques. All coronary bypass surgery cases utilised single internal mammary artery and saphenous veins as conduit. All patients were followed up for a period of 1 month. All enrolled patients completed the trial with no patients lost to follow up.

**Table 1:** **Baseline characteristics of study population**

Variables	CWC (n = 32)	AEC (n = 30)	p Value
Age (mean +/−SD)	60 +/− 35	61.5 +/− 54	0.558
Body mass index (kg/m2)	24.37 +/− 14.78	24.88 +/− 22.96	0.522
Male gender	24 (75.0)	22 (73.3)	0.881
Diabetes	22 (68.7)	19 (63.3)	0.652
Hypertension	24 (75)	22 (68.7)	0.659
IHD	31 (96.8)	26 (86.6)	0.071
Hyperlipidaemia	8 (25)	11(36.6)	0.472
Bronchial Asthma	3 (9.3)	3 (10)	0.140
CABG	30 (93.7)	24 (80.0)	0.195
Valve replacement	1 (3.1)	5 (16.7)	
CABG and valve	1 (3.1)	1 (3.3)	

All data presented as n (%) unless specified otherwise.

CWC: Conventional wire closure.

AEC: Adhesive enhanced closure.

CABG: Coronary artery bypass graft surgery.

IHD: Ischaemic heart disease.

### Primary outcome

The pain score were measured in both groups at specific interval at rest and during coughing. At rest, there were no significant differences in the postoperative pain score between the two groups at all the specific interval. Although there were trend of higher pain score in the CWC group (Table 2). However during coughing the postoperative pain score were significantly higher in the CWC group in comparison with the AEC group at 24 hours (8.04 +/− 1.43 vs. 7.00 +/− 2.09, p = of 0.042) and at 48 hours (7.12 +/− 1.60 vs. 5.27 +/− 1.971, p = 0.001). At 72 hours and 7 days post-surgery the pain score were not significantly higher in the CWC group during coughing.

**Table 2:** **Visual analogue pain score at rest and coughing**

	CWC (n = 32)	AEC (n = 30)	
Outcome variables	Mean +/− SD	Mean +/− SD	p Value
Incisional pain at rest			
Time post operative			
24 hours	5.04 +/− 2.735	4.27 +/− 2.201	0.269
48 hours	3.88 +/− 2.42	2.77 +/− 1.945	0.073
72 hours	2.46 +/− 2.319	1.81 +/− 1.877	0.269
Day 7	1.21 +/− 1.668	1.00 +/− 1.865	0.685
Incisional pain with coughing			
Time post-operative			
24 hours	8.04 +/− 1.43	7.00 +/− 2.09	0.042
48 hours	7.12 +/− 1.608	5.27 +/− 1.971	0.001
72 hours	5.31 +/ - 2.168	4.65 +/− 2.077	0.272
Day 7	3.38 +/− 1.84	2.96 +/− 2.17	0.477

CWC: Conventional wire closure.

AEC: Adhesive enhanced closure.

The levels of IL 6 were similar in both groups preoperatively. The postoperative IL 6 levels were significantly higher in the CWC group in comparison with the AEC group at 6 hours (15.987 +/− 5.528 vs. 12.809 +/− 5.593, p = 0.044), 24 hours (15. 795 +/− 5.045 vs. 12.199 +/− 5.890, p = 0.021) and 48 hours (15.770 +/− 5.526 vs. 12.056 +/− 5.046, p = 0.015) (Table 3). At 72 hours the level was also higher in the CWC group but not statistically significant.

**Table 3:** **Interleukin 6 level**

	CWC(n = 32)	AEC(n = 30)	
Outcome variables	Mean +/− SD	Mean +/− SD	p Value
Pre-op	7.44 +/− 7.86	5.53 +/− 6.01	0.353
Time post-operative			
6 hours	15.987 +/− 5.528	12.809 +/− 5.593	0.044
24 hours	15. 795 +/− 5.045	12.199 +/− 5.890	0.021
48 hours	15.770 +/− 5.526	12.056 +/− 5.046	0.015
72 hours	13.752 +/− 6.325	11.438 +/− 4.782	0.150

CWC: Conventional wire closure.

AEC: Adhesive enhanced closure.

### Secondary outcome

There were 2 deaths in the CWC group but was not statistically significant between the two groups. There was one chest reopen for bleeding in the AEC group. During the chest reopening there was no difficulty in separating the sternum as it was done 3 hours postoperatively before the adhesive set fully. Another application of adhesive was used for this patient to prevent exclusion or cross over in the study. There was more superficial sternal wound infection in the AEC group but not statistically significant (Table 4). However there was no incidence of deep sternal wound infection in both groups.

**Table 4:** **Post-operative complications**

Variables	CWC(n = 32)	AEC(n = 30)	p Value
Mortality	2 (6.25%)	0 (0%)	0.164
Chest re-open	0 (0%)	1 (3.33%)	0.313
Per-ioperative MI	1 (3.125%)	1 (3.33%)	1.000
Renal failure	2 (6.25%)	0 (0%)	0.150
Pneumonia	2 (6.25%)	0 (0%)	0.150
SSWI	1 (3.125%)	4 (13.3%)	0.161
DSWI	0 (0%)	0 (0%)	0.419

CWC: Conventional wire closure.

AEC: Adhesive enhanced closure.

SSWI: Superficial sternal wound infection.

DSWI: Deep sternal wound infection.

### Other outcome

Additional usages of analgesia were also measured during the post-operative period. Although not statistically significant, the patients in the CWC group required more additional analgesia (Table 5).

**Table 5:** **Additional analgesia used**

Variable	CWC(n = 32)	AEC(n = 30)	p Value
Yes	5 (15.6%)	2 (6.7%)	0.246

All data presented as n (%) unless specified otherwise.

CWC: Conventional wire closure.

AEC: Adhesive enhanced closure.

## Discussion

Recovery from a median sternotomy remains one of the major factors in the outcome following an open heart surgery [12]. Normally it takes up to six week for full bony union between the two hemi sternum during which time the patient can suffer from varying amount of pain and functional disability [12]. The standard closure of sternotomy involves reapproximating the two hemi sternum with multiple stainless steel wires. The configurations of the wires can be a figure of eight or a single loop. Other techniques have been introduced to improved early sternal stability and prevent complications such as sternal non-union, fracture of osteoporotic bone during closure or mediastinitis. The most popular technique is the Robicsek closure or its modification technique. In this technique essentially the 2 loops of sternal wire along the length of the sternum placed parasternally and multiple individual transverse sternal wires are loop around the longitudinal wire reapproximating the sternum [4,13]. Other techniques include using double wires loop, steel rod, plates or special clips [14,15]. However majority of these techniques requires additional introduction of wires or rod which may cause increase risk of bleeding.

In an attempt to improve and expedite the rigid stability between the two hemi sternum, addition of an adhesive type material like bone cement would be a next logical step. In orthopaedic surgery bone cement is routinely used for joint replacement surgery. Unfortunately the type of cement used has exothermic property where the temperature can reach up to 40 degree Celsius when it cures which make it unsuitable in open heart surgery due to the potential contact of the cement with the myocardial surface [16]. Furthermore this cement is normally non porous which does not allow natural osteointergration for sternal union [17]. A new biocompatible bone cement or adhesive used in our study composed of naturally occurring fatty acid and calcium carbonate derived from castor oil. The material does not exert significant heat during the setting period and takes approximately 24 hour to be fully set. When fully set there are porous networks in the adhesive which allowed host osteoblast to synthesise new bone within the networks [18].

A cadaveric study of human sternum demonstrated that the biological bone adhesive provide significant strength to the union between two hemi sternum in combination with sternal wire at 24 hours. In this study wire cerclage alone cause 2 mm lateral displacement of the two hemisternum with load as low as 400 Newton. The addition of biological bone adhesive required a load of 600 Newton or greater to cause lateral displacement [8]. The same study also demonstrated the safety of the material in human subject with SPECT evidence of osteosynthesis between the two hemisternum at 12 months [8].

In another study addition of the biological bone adhesive resulted in a significant reduction of pain at various intervals as early as 72 hours following surgery at rest and during physiological stress such as coughing [10]. As early sternal stability and pain can contribute to the respiratory function, the authors have also demonstrated significant improvement in inspiratory capacity at 72 hours following surgery in the adhesive enhanced closure group. The rapid return to baseline health related quality of life was demonstrated as early as 3 weeks in the adhesive enhanced closure. The risk for sternal complication is increasing in the current cardiac surgery population which include older age, obesity, osteoporosis, diabetes, COPD and utilisation of bilateral internal mammary artery [19,20]. This adjunct technique of achieving early bony union most likely will benefits these high risk groups of patients. Early bony union will undoubtedly reduce the stress on the bone from the sternal wire hence reduce risk of complication of sternal non-union. A recent study has demonstrated the benefits of the adhesive enhanced sternal closure in this group of patients. In the study, high risk group such as the elderly experienced no severe pain, improved respiration and achieved early good quality of life [11].

In our study we have focused mainly on the pain experienced by the patients. To be more objective we have utilised the visual analogue score and Interleukin 6 (IL 6) as pain biomarker to quantify pain. In our study, the adhesive enhanced group demonstrated significant reduction of incisional pain score during coughing postoperative at 24 and 48 hours. These findings can be explained as the adhesive takes approximately 24 hours to be fully set. In our study the pain score were not significantly higher at rest. It has been shown that after a standard method of closure with wires alone, micro movement occurred between the two hemisternum during physiological distraction such as coughing [21]. The micro movement can cause discomfort for the patients and potentially delayed the healing and increase the risk of sternal infection. The lack of micro movement at rest explained similar pain score experienced at rest by the two groups. At 72 hours the pain score was less in the adhesive group but was not statistically significant. In Fedak et al. study the pain score at 72 hours was significantly reduced in the adhesive enhanced group. This could be explained by our dual analgesia protocol of non-opioid and opioid in both groups.

In our study the absolute value of pain reduction although significant is small ranging from 0.6 to 1.91. These values are lower than the predicted value of 3 during the planning of the study and also lower than a previous study [10]. As pain is a subjective measure with significant interpersonal variability and depends on the type and reliability of the pain scale used, a more objective assessment of pain with a biochemical marker was done to substantiate the advantage of adhesive enhanced closure. IL 6 a biomarker used in this study is an inflammatory cytokine that has an important role in the physiology of nociception and the pathophysiology of pain. Its receptor gp80, and its transmembranous signal transducer gp 130 are up-regulated in periphery nerves, dorsal root ganglia and spinal cord during experimental pain [22]. In human IL 6 is among the most sensitive indicator of the degree of surgical stress [23]. Previous studies have shown a reduction in IL 6 level post thoracotomies when there were reduction in pain due to additional pain controlled methods such as additional NSAID and TENS [24,25]. We have not included other set of biomarkers such as CRP TNF or other Interleukin due to the previous studies explained earlier which suggest IL 6 is the most accurate biomarker for pain. Furthermore the main aim of the study is pain assessment and not surgical stress. Our data demonstrated in comparison with conventional sternal closure, the adhesive enhanced closure was associated with significant lower level of IL 6 at 6, 24 and 48 hours post-surgery. In our study the invasiveness of surgery was similar in both groups. Thus as an indirect objective marker of pain, lower level of IL 6 released in the AEC group confirmed the advantage of the biological bone adhesive as an adjunct to conventional sternal closure. The porosity of the bone adhesive which allows osteointergration implied that the biological bone adhesive does not reduced inflammatory response which can contribute to lower IL 6 level. Indirectly, the lower level of IL 6 in the AEC group may purely due to reduced pain response.

In our study the utilisation of additional analgesia were lower in the treatment group but was not statistically significant which did not replicate the findings from Fedak et al. study where the level of overall opioid usage were statistically less in the adhesive enhanced closure group. The reason for this finding may be due to the fact that our standard post-operative analgesia protocol which include oral opioid. All the additional analgesia used in our study was opioid base infusion.

In the study there were more superficial sternal wound infections in the treatment group but not statistically significant. Although occasionally superficial sternal wound infection can progress to deep sternal wound infection, none of the patients developed deep sternal wound infection. All the infections were successfully treated with intravenous antibiotics. We therefore believe that the utilisation of the bone adhesive did not contribute to the superficial wound infection. There were no other significant complications from the utilisation of the bone adhesive in our study. In the literature there has been no adverse reaction reported with the utilisation of the bone adhesive. However possible adverse reaction or limitation include allergic reaction, excessive spillage into mediastinum compressing grafts after approximation of sternum, possible arrhythmias from the slight heat generated from the adhesive whilst it set. Other limitation include the necessity of an oscillating saw should the case require a chest reopen after 24 hour when the adhesive is fully set.

### Study limitation

In our study, several limitations were identified. The small number in size could have contributed to the non-significant results at 72 hour following surgery. Inclusion of multiple types of open heart surgery procedures which involves sternotomy might have influenced some of our findings. Our follow up is only for one month with no measurement of quality of life which may have added further strength to the study. Our standard dual analgesia protocol of non-opioid and opioid could have contributed to some of non-significant results when compare to other studies. Utilisation of only non-opioid analgesia to see reduction of opioid usage would be useful in future studies.

## Conclusion

The large reduction in pain score from other studies has not been replicated in our study. The addition of biological bone adhesive to the standard method of sternal closure resulted in marginal significant reduction of pain although confirmed by the reduction of pain biomarker released. Due to the marginal improvement of pain together with similar requirement of analgesia its clinical significant maybe doubtful base on our current study. As a new product the safety of its use was shown in several studies including ours. Limitations for its use would be the additional cost it incurs and the modest improvement in pain score base from our study. Further larger multi-centre trial with longer follow-up together with cost benefits analysis to confirm its clinical benefits further is needed before this technique can widely adopted.

## Consent

Written informed consent was obtained from the patient for the publication of this report and any accompanying images.
